# Laboratory- and field-based performance-predictions in cross-country skiing and roller-skiing

**DOI:** 10.1371/journal.pone.0256662

**Published:** 2021-08-24

**Authors:** Rune Kjøsen Talsnes, Guro Strøm Solli, Jan Kocbach, Per-Øyvind Torvik, Øyvind Sandbakk

**Affiliations:** 1 Meråker High School, Trøndelag County Council, Steinkjer, Norway; 2 Department of Sports Science and Physical Education, Nord University, Bodø, Norway; 3 Department of Neuromedicine and Movement Science, Centre for Elite Sports Research, Norwegian University of Science and Technology, Trondheim, Norway; Universita degli Studi di Verona, ITALY

## Abstract

The purpose of the present study was to investigate how various laboratory- and field-based tests predict on-snow cross-country (XC) skiing and roller-skiing performance. Thirty-three national-level male XC skiers (19.0±2.5 years, maximal oxygen uptake [VO_2max_] 70.8±4.7 mL·min^-1^·kg^-1^) performed a 13.6-km roller-ski skating competition tracked by a global positioning system (GPS), which together with individual distance International Ski Federation (FIS) points was used to assess their performance level. On separate days, time in a 6.4-km uphill running time-trial (RUN-TT) and 1.3-km uphill roller-ski double-poling time-trial (DP-TT) was measured in the field and performance indices determined while running and roller-ski skating in the laboratory. The mean finishing times for the RUN-TT and the DP-TT showed moderate to large correlations with distance FIS points and performance in the roller-ski skating competition (r = 0.56–0.72; all p<0.05). RUN-TT was more strongly correlated with distance FIS points than DP-TT (r = 0.72 versus 0.56; p<0.05). Performance indices and VO_2max_ in incremental running and roller-ski skating in the laboratory showed large to very large correlations with distance FIS points and roller-skiing performance (r = 0.50–0.90; all p<0.05). Performance indices and VO_2max_ in running tended to be more strongly correlated with roller-skiing performance than corresponding values obtained while roller-ski skating (all p<0.10). The present findings suggest that both laboratory performance indices and field-based performance tests provide valid predictions of XC skiing and roller-skiing performance in a heterogeneous group of male XC skiers, with test values obtained in running tending to be more strongly correlated with XC skiing performance than those found for technique-specific modalities on roller skis. However, more sophisticated and mode-specific testing might be required for more homogenous groups of elite XC skiers.

## Introduction

Cross-country (XC) skiing is an endurance sport combining upper- and lower-body work to cross varied terrain in competitions lasting from ~3 min to ~2 h [1]. The demands of XC skiing require an exceptionally high aerobic energy turnover combined with efficient skiing techniques in the classical and skating styles, while anaerobic power, strength, and speed play additional roles in determining performance [1]. Furthermore, roller skiing, as an off-snow equivalent to XC skiing, has evolved from being only a training mode in the summer months into a competitive sport. However, the physiological requirements and performance determinants in competitive roller skiing are currently unexplored.

XC skiers have always shown some of the highest reported values of maximal oxygen uptake (VO_2max_) [2–6], which are traditionally measured in running. However, the development of customized ski-specific treadmills and ergometers has provided the opportunity to examine physiological capacities using sport-specific modes. Accordingly, performance indices (e.g., peak speed and time to exhaustion) and peak oxygen uptake (VO_2peak_) in various roller skiing sub-techniques are typically used for monitoring athletes, as they have been strongly correlated with XC skiing performance [7–15], and in particular, with performance in uphill terrain [10, 12, 15]. Moreover, the ability to efficiently convert metabolic energy into external work and speed (i.e., gross efficiency) has been correlated with XC skiing performance [10, 16]. However, it has not yet been examined whether these technique-specific tests provide more valid predictions for XC skiing and roller skiing performance than traditional running tests.

Field-based performance tests within the most common training modes, roller skiing and running, are commonly employed to monitor training adaptations and performance development in XC skiers [17]. The advantage of employing field-based tests in the training process is their high ecological validity combined with less need for expensive equipment and trained test personnel. Such tests are often performed in steep uphill terrain, challenging the athlete’s maximal aerobic power and avoiding technical difficulties and thereby obtaining higher test-retest reliability [18]. Moreover, field-based tests in roller-skiing are often performed exclusively by using the double-poling technique to specifically target the upper-body strength and endurance required for effective double-poling in XC skiing [8, 14, 19–21]. Although field-based tests are commonly used in XC skiing today, their significance for performance and association with laboratory-based capacities are still understudied.

The understanding of how different laboratory- and field-based tests predict performance is essential for improving coaches’ and athletes’ ability to optimize the design and systematic use of different tests in their training process. Therefore, the purpose of the present study was to investigate how various laboratory- and field-based tests predict on-snow XC skiing and roller-skiing performance. It was hypothesized that tests performed in technique-specific modalities (i.e., roller skiing) would demonstrate stronger relationship with XC skiing and roller-skiing performance than comparable tests performed in running.

## Methods

### Participants

Thirty-three (24 junior and 9 senior) national-level male XC skiers volunteered to participate in the study. All athletes were students at a Norwegian High School or University with a specialized study program for XC skiing. The mean ± standard deviation (SD) characteristics of the group were: age, 19.0±2.5 years; body mass, 71.4±7.4 kg; height, 181.3±6.5 cm; VO_2max_, 70.8±4.7 mL·min^-1^·kg^-1^. The study was approved by the Norwegian Centre for Research Data (NSD). All athletes gave written informed consent in accordance with the Declaration of Helsinki, and parental consent was provided for athletes aged <18 years (n = 8).

### Overall design

All athletes performed a roller-ski skating competition (13.6-km time-trial [TT]) while being tracked by a global positioning system (GPS). The race course was mapped with a coupled GPS and barometer to provide a valid course and elevation profile. The XC skiing course was further divided into uphill, intermediate, and downhill terrain sections. Data from the roller-ski skating competition and individual distance FIS points were used to assess the athletes overall and terrain-specific performance levels. Within a three-week period from the roller-ski skating competition, the athletes completed a 6.4-km uphill running TT (RUN-TT, n = 33) and 1.3-km uphill roller-ski double-poling TT (DP-TT, n = 33) in the field, as well as tests of performance indices while running (n = 35) and roller-ski skating (n = 38) in the laboratory.

### Roller-ski skating competition and field-based tests

**Fig 1:**
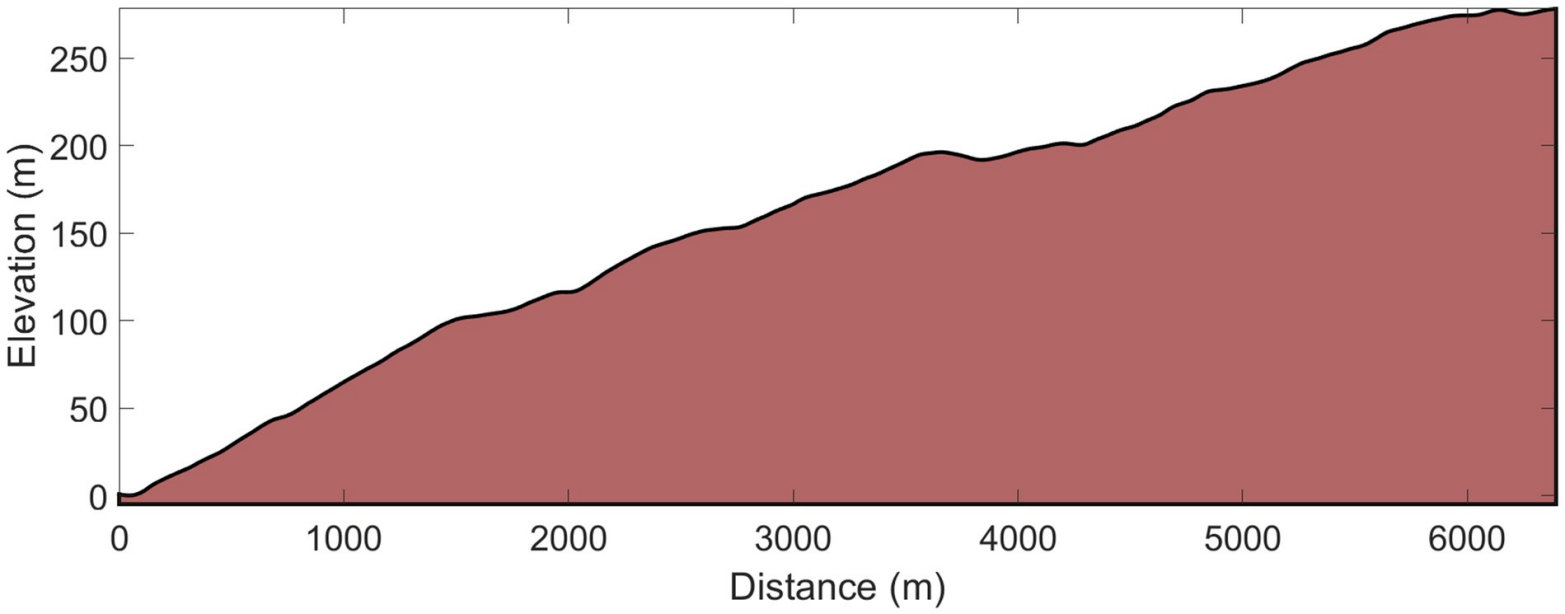
Course and elevation profile of the 6.4-km uphill running time-trial.

**Fig 2:**
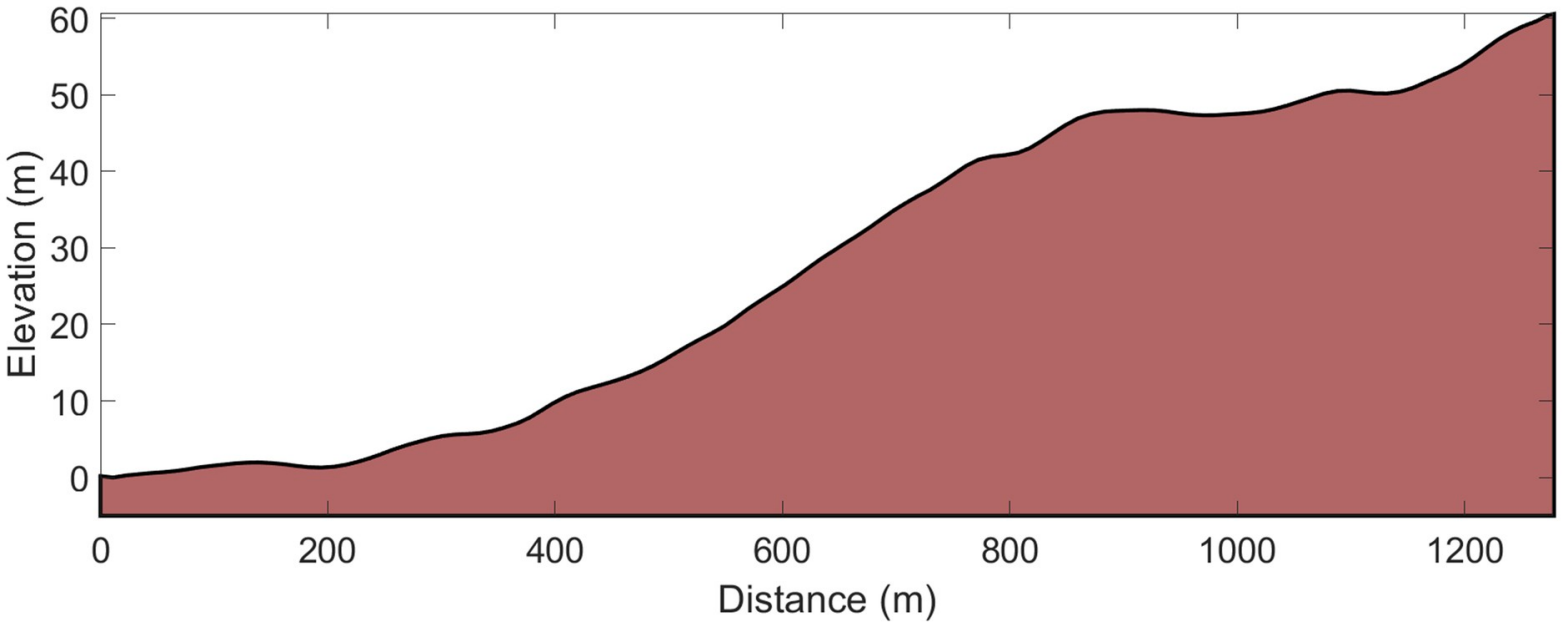
Course and elevation profile of the 1.3-km uphill double-poling time-trial.

Prior to both the roller-ski skating competition and field-based tests, the athletes performed ~30-min of self-selected warm-up. Performance times were recorded using two synchronized watches and the Racesplitter timekeeping system (Makalu Logistics Inc., Fontana, CA, USA) with a 30 s starting interval between each athlete. Course and elevation profiles (Figs 1 and 2) were measured with an integrated GPS and barometer using a Garmin Forerunner 920 XT (Garmin Ltd., Olathe, KS, USA). For the roller-ski skating competition and the DP-TT, all athletes used the same type of skating and classical roller-skis with standard category two wheels (IDT Sports, Lena, Norway). The weather conditions were stable and similar for all three test days being partly cloudy, with ambient air temperatures of 12–15°C, low and stable wind, and relative humidity varying between 70% and 80%. All athletes were instructed to perform standardized training loads (low-intensity sessions of <1.5 h) over the final two days prior to testing and not consume any caffeinated beverages 24 h before and large meals 2 h before the tests.

#### GPS analysis

**Fig 3:**
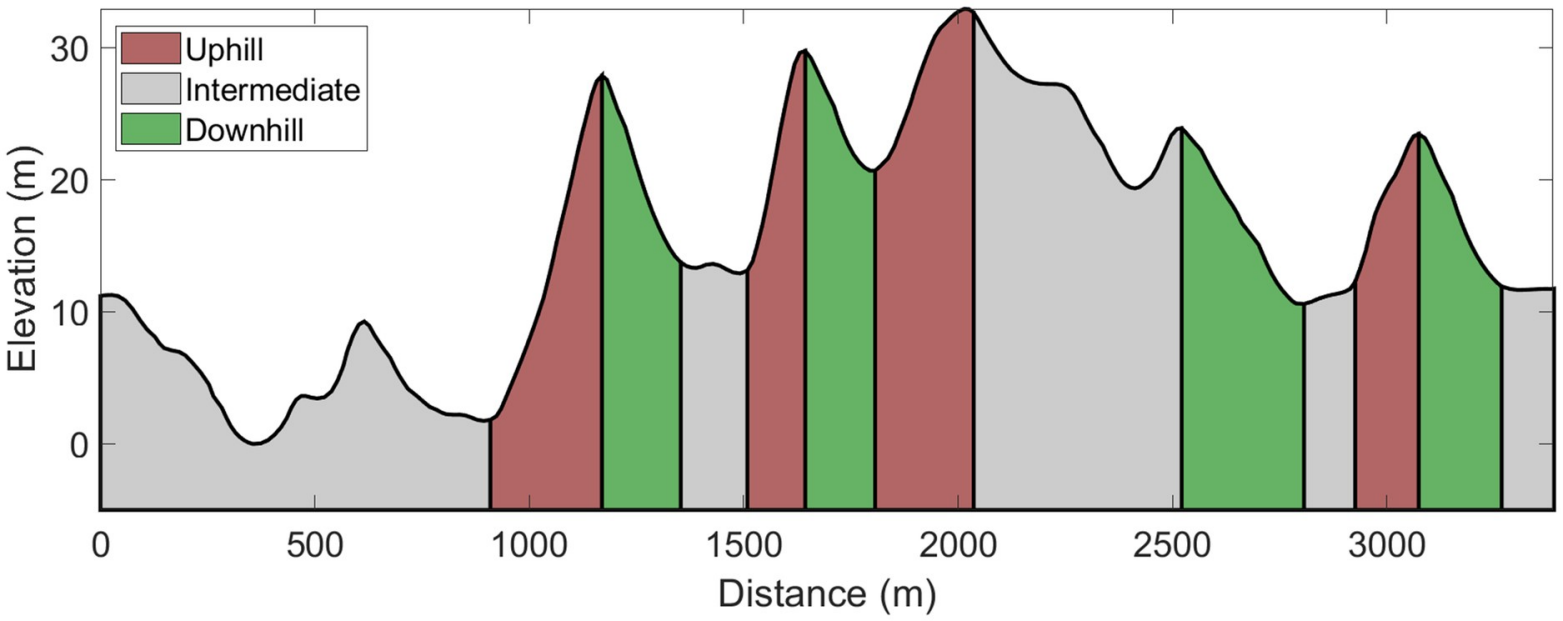
Course and elevation profile of the 13.6-km roller-ski skating competition, including different sections of terrain.

The roller-ski skating competition was performed at an international FIS-regulated race course consisting of 4×3.4-km laps (Fig 3). All athletes were tracked by a wrist-worn Garmin Forerunner 920 XT GPS watch, which collected position data at a 1-Hz sampling rate. All GPS watches were turned on at least 20-min before the test to optimize GPS accuracy. Thereafter, data for all the athletes were adapted to the defined course and elevation profile (reference course) by fitting each competitor’s GPS track to points along the reference course. This methodology has previously been described in detail [22] and the GPS device used in the current study validated against higher-accuracy GPS devices [23]. The reference course was further divided into uphill, intermediate, and downhill terrain. The classification of different terrain sections was adopted from the FIS homologation manual for XC skiing race courses [24]. A section boundary was defined where a change between positive and negative gradients in the course profile occurred. Terrain sections with a climb of >10 m and gradient of >5% were classified as uphill sections, while sections with a descent of >10 m and negative gradient of >5% were classified as downhill sections. The remaining sections were classified as intermediate terrain, including short uphill and downhill sections interspersed with flat sections.

### Laboratory testing

#### Running test (day 1)

Following a 10-min low-intensity warm-up running (60–72% of maximal heart rate [HR_max_]), the athletes performed one 5-min submaximal stage, running at a 10.5% incline and at the same absolute speed (8 km·h^-1^) to measure physiological and perceptual responses. After 2-min of recovery, a time to exhaustion (TTE) test was performed to determine VO_2max_ and performance measured as TTE and peak speed [11]. The test was conducted at the same incline, with a starting speed of 9 km·h^-1^ and thereafter, 1-km·h^-1^ increases in speed every minute until exhaustion.

#### Roller-ski skating test (day 2)

After the same warm-up procedure as for test-day 1, all athletes performed two 5-min submaximal stages at a 5% incline and the same absolute speed (12 and 14 km·h^-1^, respectively), with a 1-min recovery in between, during roller-ski skating. Only the 12 km·h^-1^ submaximal stage was used for analyses. Submaximal testing was followed by a 5-min recovery prior to an incremental test for the determination of VO_2peak_, TTE and peak speed [11]. In roller-ski skating, VO_2peak_ was used because, on average, the group reached 95% of their running-VO_2max_ in this technique-specific modality. The test was performed at a similar incline, with a starting speed of 14 km·h^-1^. Inclination was kept constant, while the speed was increased by 2 km·h^-1^ per min up to 20 km·h^-1^ and subsequently by 1 km·h^-1^ per min until exhaustion. The athletes were instructed to use the skating G3 sub-technique during both the submaximal and TTE test. All athletes were familiarized with treadmill roller-ski skating and the test protocol before testing.

For all submaximal testing, respiratory recordings were collected between the third and fourth minute of each 5-min stage, and heart rate (HR) was defined as the average over the last 30 s. The rating of perceived exertion (RPE), using the 6–20-point Borg scale, and blood lactate levels were assessed immediately after completing each stage. In addition, gross efficiency was measured for the submaximal roller-ski skating stage and defined as the ratio of work and metabolic rate [25]. During the TTE tests, respiratory variables and HR were measured continuously, and VO_2max_ was defined as the average of the two highest consecutive 30 s measurements. HR_max_ was defined as the highest 5-s HR measurement during each test, whereas RPE was determined immediately after each test and blood lactate levels ~1 min after.

#### Instruments and materials

Running tests were performed on a 2.5 x 0.7-m motor-driven treadmill and roller-ski skating tests on a 3.5 x 2.5-m treadmill (RL 2500 and RL 3500E, Rodby, Vänge, Sweden). The treadmill belt consisted of a non-slip rubber surface, allowing the athletes to use their own poles with special carbide tips. All athletes used the same pair of skating roller-skis with standard category 2 wheels (IDT Sports, Lena, Norway). The athletes were always secured with a safety harness hanging from the ceiling, connected to the safety brake system of the treadmill. Rolling friction force (Ff) was measured in a towing test as previously described by [25], providing an average μ value of 0.017, which was used to calculate work rate.

Respiratory variables were measured using open-circuit indirect calorimetry (Oxycon Pro, Jaeger GmbH, Hoechberg, Germany). The instruments were calibrated against ambient air conditions and certified gases of known concentrations (O_2_ 15.0%; CO_2_ 5.0%) before each test session. The flow transducer (Triple V, Jaeger GmbH, Hoechberg, Germany) was calibrated using a 3-L high-precision calibration syringe (Calibration Syringe D, SensorMedics, Yorba Linda, CA, USA). HR was continuously measured with a Garmin Forerunner 920 XT. Blood lactate levels were measured using the stationary Biosen C-Line lactate analyser (Biosen, EKF Industrial Electronics, Magdeburg, Germany) in 20 μL of blood taken from the fingertip. The athletes’ body masses and heights were measured using a medical weight and stadiometer (Seca model 708, Seca GmbH, Hamburg, Germany).

### Statistical analysis

Continuous variables are presented as mean ± SD and were examined for the assumption of normal distribution before analysis using a Shapiro–Wilk test and visual inspection of Q–Q plots and histograms. Data were processed and analyzed using IBM SPSS Statistics version 26 software for Windows (SPSS Inc., Chicago, IL, USA) and Office Excel 2016 (Microsoft Corporation, Redmond, WA, USA). Correlation analyses between XC skiing performance, field- and laboratory-based tests were conducted using the parametric Pearson’s or non-parametric Spearman’s correlation coefficients. The strength of the correlations was interpreted according to Hopkins et al. [26] r<0.1 = trivial, 0.1–0.3 = small, 0.3–0.5 = moderate, 0.5–0.7 = large, 0.7–0.9 = very large, 0.9 = nearly perfect, and 1.0 = perfect. To statistically compare the strength of correlations, the hypothesis tests for comparisons of correlations according to Chen and Popovich [27] were applied. In addition, block-wise multiple regression analyses, with models consisting of 1–3 independent variables (field- or laboratory based tests) and XC skiing performance as the dependent variable was performed. Alpha values of <0.05 determined the statistical significance level, and alpha values of 0.05–0.1 were considered as trends.

## Results

The overall finishing time for the roller-ski skating competition was 34:22±02:03 min, during which the athletes generally employed a positive lap-to-lap pacing pattern (gradual reduction in speed from lap 1 to lap 3, although a small increase in speed was seen in lap 4; Table 1). The relative time spent in uphill, intermediate, and downhill terrain was 38%, 44%, and 18% of the overall time, respectively. The overall time and the time spent in uphill, intermediate, and downhill terrains demonstrated large to very large correlations with distance FIS points (r = 0.80, 0.79, 0.78 and 0.60, respectively; all p<0.001).

**Table 1:** Distance, time, and speed for a 6.4-km uphill running time-trial, a 1.3-km uphill double-poling time-trial and a 13.6-km roller-ski skating competition in a group of national-level male cross-country skiers (n = 33).

	Distance (m)	Time (s)	Speed (m·s^-1^)
**6.4-km uphill running TT**			
Overall	6400	1695 ± 123 (472)	3.8 ± 0.3 (1.1)
**1.3-km uphill DP TT**			
Overall	1280	269 ± 20 (86)	4.8 ± 0.3 (1.5)
**13.5-km roller-ski skating competition**			
Overall	13560	2062 ± 134 (564)	6.6 ± 0.4 (1.8)
Lap 1	3390	504 ± 29 (123)	6.8 ± 0.4 (1.7)
Lap 2	3390	518 ± 35 (168)	6.6 ± 0.4 (2.1)
Lap 3	3390	524 ± 37 (146)	6.5 ± 0.5 (1.9)
Lap 4	3390	518 ± 37 (147)	6.6 ± 0.5 (1.9)
Uphill terrain	3092	789 ± 77 (325)	4.0 ± 0.4 (1.7)
Intermediate terrain	7164	898 ± 47 (205)	8.0 ± 0.4 (1.9)
Downhill terrain	3304	377 ± 16 (70)	8.8 ± 0.4 (1.6)

Data are presented as mean ± SD (range).

SD = standard deviation; TT = time-trial; DP = double-poling.

**Fig 4:**
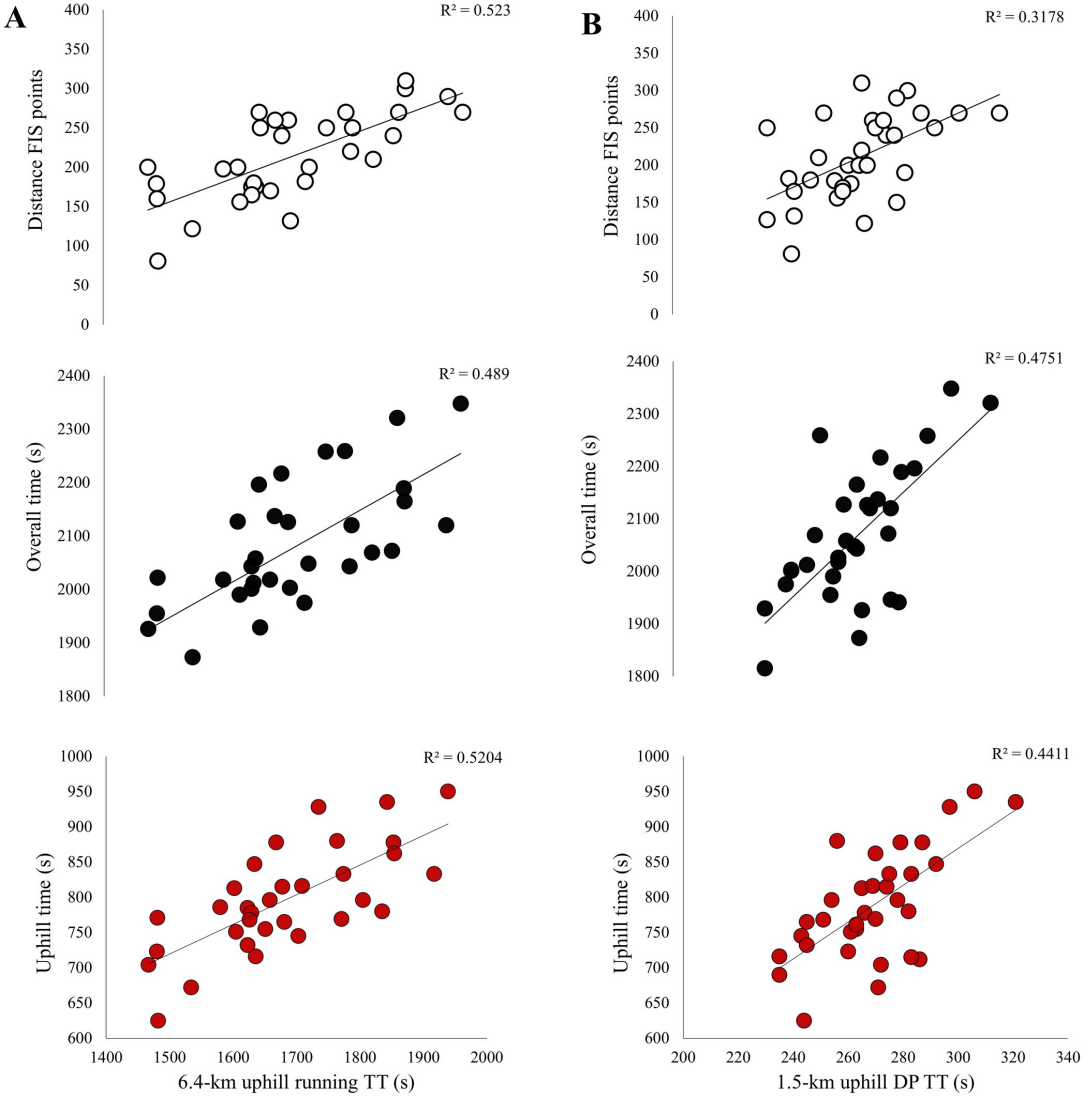
Relationship of distance FIS points and overall and terrain-specific performance in the 13.6-km roller-ski skating competition to (A) 6.4-km uphill running time-trial and (B) 1.3-km uphill double-poling time-trial in a group of national-level male cross-country skiers (n = 33). Presented with individual data points and trend lines based on linear regression.

The mean finishing times for the RUN-TT and DP TT were 28:15±02:07 min and 04:19±00:18 min, respectively. Correlations between the field-based tests and distance FIS points, as well as the roller-ski skating competition are presented in Fig 4. Both field-based tests showed moderate to large correlations with distance FIS points in addition to overall performance and performance in uphill, intermediate, and downhill terrain in the roller-ski skating competition (all p<0.05). The RUN-TT was more strongly correlated with distance FIS points (p = 0.05) and tended to be more strongly correlated with uphill performance (p = 0.09) than DP-TT, whereas DP-TT was more strongly correlated with performance in downhill terrain in the roller-ski skating competition than RUN-TT (p = 0.02).

Laboratory-based tests are presented in Table 2, and correlations between parameters determined in these tests and performance in the two field-based tests are shown in Table 3. Peak speed and body-mass-normalized VO_2max_ in running were more strongly correlated with RUN-TT performance than corresponding values obtained while roller-ski skating (all p<0.05), whereas body mass and absolute VO_2peak_ in roller-ski skating were more strongly correlated with DP-TT performance than similar values in running (both p<0.05).

**Table 2:** Descriptive statistics for laboratory-based tests running and roller-ski skating in a group of national-level male cross-country skiers (n = 33).

	Running	Roller-ski skating
**Submaximal test**	**(8 km·h** ^**-1**^ **)**	**(12 km·h** ^**-1**^ **)**
VO_2_ in %VO_2max_	66.7 ± 5.1 (22.0)	69.0 ± 6.1 (23.8)
Heart rate in %HR_max_	80.0 ± 5.4 (22.1)	84.0 ± 5.7 (23.0)
Borg (6–20)	11.8 ± 1.7 (6.0)	11.8 ± 1.5 (6.0)
Blood lactate (mmol·L^-1^)	2.00 ± 0.67 (3.04)	2.67 ± 0.98 (3.84)
Gross efficiency (%)	NaN	14.0 ± 0.5 (2.9)
**TTE test**		
VO_2max_ (L·min^-1^)	5.05 ± 0.56 (2.38)	4.89 ± 0.66 (3.03)
VO_2max_ (mL·min^-1^·kg^-1^)	70.8 ± 4.7 (21.4)	68.1 ± 5.2 (17.6)
HR_max_ (beats·min^-1^)	196 ± 10 (39)	194 ± 9.5 (41)
Blood lactate (mmol·L^-1^)	11.49 ±2.07 (9.46)	11.55 ± 1.70 (7.22)
Borg (6–20)	19.0 ± 0.8 (3.0)	18.9 ± 0.8 (3.0)
TTE (s)	402 ± 61 (280)	328 ± 76 (360)
Speed_peak_ (km·h^-1^)	15.7 ± 1.0 (4.5)	22.4 ± 1.3 (7.0)

Data are presented as mean ± SD (range).

SD = standard deviation; VO_2_ = oxygen uptake; HR_max_ = maximal heart rate; TTE = time to exhaustion; Speed_peak_ = peak treadmill speed; VO_2max_ = maximal oxygen uptake; HR_peak_ = peak heart rate; VO_2peak_ = peak oxygen uptake.

**Table 3:** Correlations between the main laboratory performance indices and field-based tests in a group of national-level male cross-country skiers (n = 33).

	RUN-TT (s)	DP-TT (s)
**Running TTE test**		
VO_2max_ (L·min^-1^)	-0.38*	-0.73**
VO_2max_ (mL·min^-1^·kg^-1^)	-0.82**	-0.58**
TTE (s)	-0.80**	-0.73**
Speed_peak_ (km·h^-1^)	-0.83**	-0.74**
**Roller-ski skating TTE test**		
VO_2peak_ (L·min^-1^)	-0.29	-0.67**
VO_2peak_ (mL·min^-1^·kg^-1^)	-0.65**	-0.54**
TTE (s)	-0.64**	-0.62**
Speed_peak_ (km·h^-1^)	-0.65**	-0.67**

RUN-TT = running time-trial; DP-TT = double-poling time-trial; VO_2max_ = maximal oxygen uptake; TTE = time to exhaustion; Speed_peak_ peak treadmill speed; VO_2peak_ = peak oxygen uptake.

*p<0.05;

**p<0.01.

**Fig 5:**
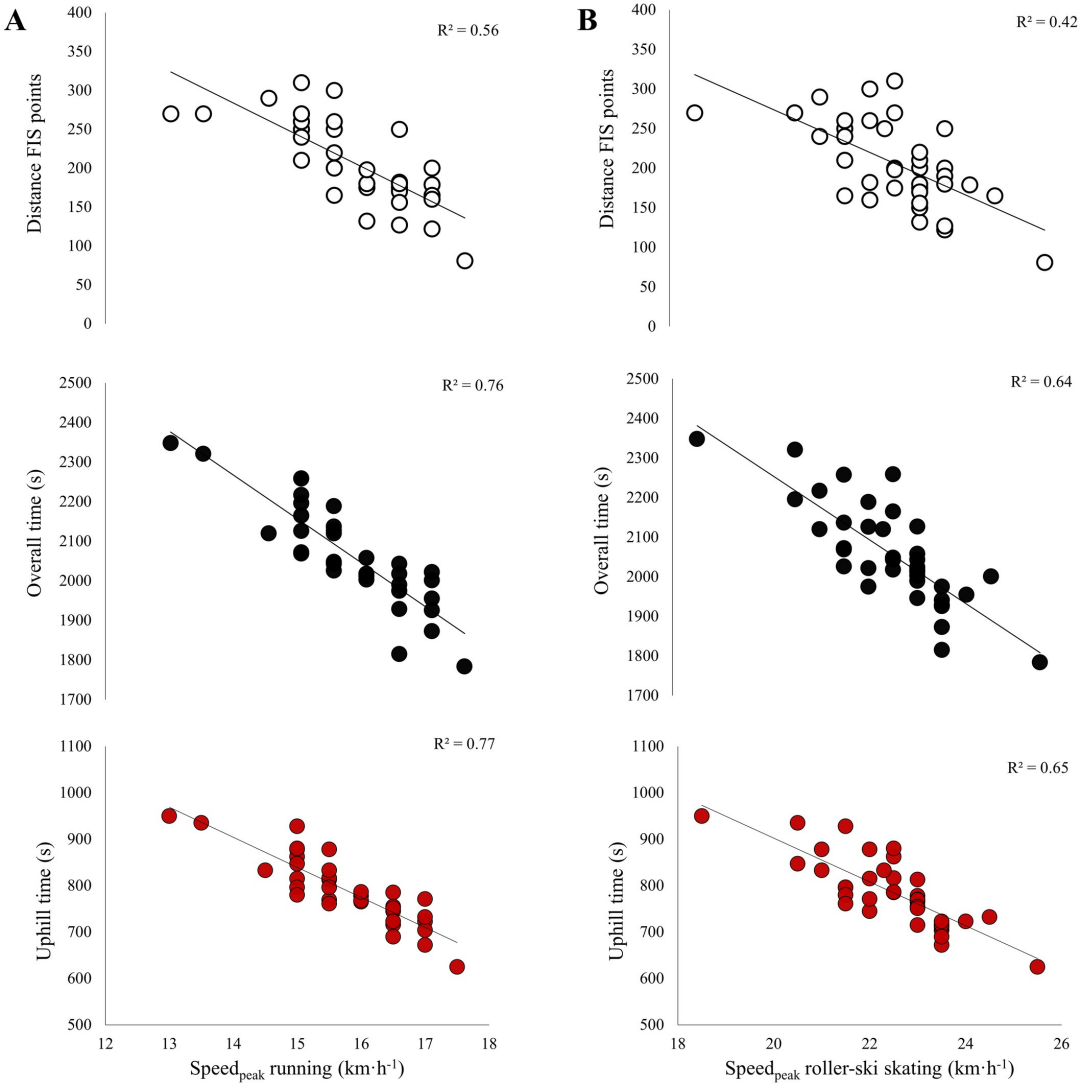
Relationship of distance FIS points and overall and terrain-specific performance in a 13.6-km roller-ski skating competition to (A) peak speed (Speed_peak_) in running (B) peak speed (Speed_peak_) in roller-ski skating during laboratory testing among a group of national-level male cross-country skiers (n = 33). Presented with individual data points and trend lines based on linear regression.

**Fig 6:**
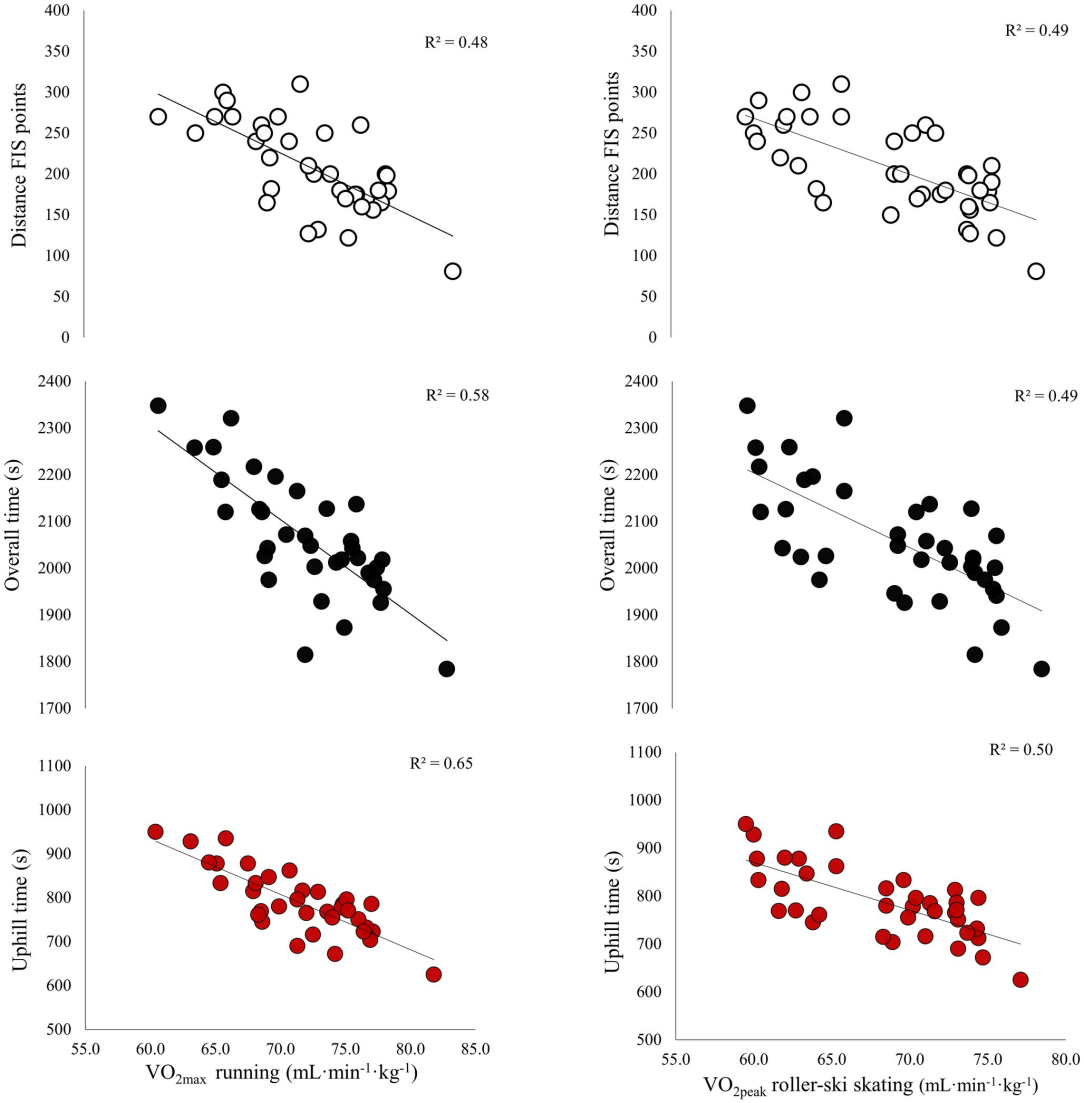
Relationship of distance FIS points and overall and terrain-specific performance in the 13.6-km roller-ski skating competition to (A) maximal oxygen uptake (VO_2max_) in running and (B) peak oxygen uptake (VO_2peak_) in roller-ski skating during laboratory testing among a group of national-level male cross-country skiers (n = 33). Presented with individual data points and trend lines based on linear regression.

Only trivial to small correlations were found between body mass and body mass index versus distance FIS points, as well as overall and terrain-specific performance in the roller-ski skating competition. Correlations between laboratory performance indices and distance FIS points and performance in the roller-ski skating competition are presented in Table 4 and Figs 5 and 6. Peak speed and VO_2max_ in running and roller-ski skating showed large to very large correlations with distance FIS points, overall performance, and performance in all terrains in the roller-ski skating competition (all p<0.01). Body-mass-normalized VO_2max_ in running and roller-ski skating were more strongly correlated with uphill performance than with performance in intermediate and downhill terrains in the roller-ski skating competition (all p<0.05). Peak speed tended to be more strongly correlated with distance FIS points, as well as overall and uphill performance in the roller-ski skating competition for running than for roller-ski skating (all p<0.10). Absolute and body-mass-normalized VO_2max_ values in running tended to be more strongly correlated with uphill performance in the roller-ski skating competition than VO_2peak_ obtained during roller-ski skating (both p<0.10).

**Table 4:** Correlations of field- and laboratory-based tests with distance FIS points as well as with overall performance and performance in different terrains of a 13.6-km roller-ski skating competition in a group of national-level male cross-country skiers (n = 33).

	FIS points	Roller-ski skating competition			
		Overall time (s)	Uphill terrain (s)	Intermediate terrain (s)	Downhill terrain (s)
**Field tests**					
1.3-km uphill DP TT (s)	0.56**	0.69**	0.66**	0.66**	0.66**
6.4-km uphill running TT (s)	0.72**	0.70**	0.72**	0.66**	0.43*
**Anthropometrics**					
Body mass (kg)	-0.12	-0.20	-0.10	-0.31	-0.25
Body mass index (kg·m^-2^)	-0.16	-0.14	-0.08	-0.19	-0.18
**Submaximal test running (8 km·h** ^**-1**^ **)**					
VO_2_ in %VO_2max_	0.74**	0.79**	0.79**	0.72**	0.70**
Heart rate in %HR_max_	0.64**	0.69**	0.63**	0.71**	0.62**
Borg (6–20)	0.44**	0.32	0.30	0.36	0.20
Blood lactate (mmol·L^-1^)	0.41*	0.49**	0.44**	0.56**	0.38*
**TTE test running**					
VO_2max_ (L·min^-1^)	-0.57**	-0.66**	-0.70**	-0.70**	-0.62**
VO_2max_ (mL·min^-1^·kg^-1^)	-0.69**	-0.76**	-0.80**	-0.65**	-0.64**
TTE (s)	-0.74**	-0.87**	-0.90**	-0.80**	-0.70**
Speed_peak_ (km·h^-1^)	-0.75**	-0.87**	-0.88**	-0.80**	-0.70**
**Submaximal test roller-ski skating (12 km·h** ^**-1**^ **)**					
VO_2_ in % VO_2peak_	0.61**	0.73**	0.74**	0.64**	0.69**
Heart rate in %HR_peak_	0.54**	0.71**	0.73**	0.62**	0.58**
Borg (6–20)	0.20	0.30	0.25	0.34	0.30
Blood lactate (mmol·L^-1^)	0.49*	0.57**	0.58**	0.52**	0.50**
Gross efficiency (%)	-0.09	-0.27	-0.30	-0.19	-0.26
**TTE test roller-ski skating**					
VO_2peak_ (L·min^-1^)	-0.51**	-0.57**	-0.50**	-0.62**	-0.50**
VO_2peak_ (mL·min^-1^·kg^-1^)	-0.70**	-0.70**	-0.71**	-0.63**	-0.60**
TTE (s)	-0.63**	-0.79**	-0.80**	-0.72**	-0.64**
Speed_peak_ (km·h^-1^)	-0.65**	-0.80**	-0.81**	-0.73**	-0.69**

FIS, International Ski Federation; DP, double-poling; TT, time-trial; VO_2_ = oxygen uptake; HR_max_ = maximal heart rate; TTE = time to exhaustion; Speed_peak_ = peak treadmill speed; VO_2max_ = maximal oxygen uptake; HR_peak_ = peak heart rate; VO_2peak_ = peak oxygen uptake.

*p<0.05;

**p<0.01.

Physiological and perceptual responses during submaximal running and roller-ski skating, including %VO_2max_, %HR_max_, and blood lactate levels, showed large to very large correlations with distance FIS points, overall performance, and performance in different terrains in the roller-ski skating competition (all p<0.05). Whether physiological values were obtained in running or roller-ski skating did not influence the strength of the correlations. Submaximal RPE in both modalities and gross efficiency in roller-ski skating revealed only trivial or small correlations with distance FIS points or overall and terrain-specific XC skiing performance.

Regression analyses, with laboratory- and field-based tests as independent variables, resulted in the following equations as the best predictions for distance FIS points and overall performance in the roller-skiing competition:

$$1.\;\text{Distance}\;\text{FIS}\;\text{points}=261+0.17\;\cdot \;\text{RUN-TT}\;\left(\text{s}\right)-5.00\;\cdot \;{\text{roller-ski-VO}}_{2\text{peak}}\;(\text{mL}\cdot {\text{min}}^{-1}\cdot {\text{kg}}^{-1})$$

Both factors included in the equation significantly contributed to the model (p<0.01) and explained 63% of the variance in distance FIS points.


$$2.\;\text{Overall}\;\text{performance}=4900-0.30\;\cdot \;\text{RUN-TT}\;\left(\text{s}\right)-100\;\cdot \;{\text{running-VO}}_{2\text{max}}\;(\text{mL}\cdot {\text{min}}^{-1}\cdot {\text{kg}}^{-1})–10\;\cdot \;{\text{running-speed}}_{\text{peak}}\;(\text{km}\cdot {\text{h}}^{-1})$$

VO_2max_ and peak speed in running contributed significantly to the model (both p<0.01), and there was a trend for RUN-TT to contribute (p = 0.06). The model explained 82% of the variance in overall performance in the roller-ski skating competition.

## Discussion

The present study investigated how various laboratory- and field-based tests predict XC skiing and roller-skiing performance in a group of national-level male XC skiers. The main findings were as follows: (1) both uphill RUN-TT and uphill DP-TT performance showed moderate to large correlations with distance FIS points and roller-skiing performance; (2) RUN-TT performance was more strongly correlated with distance FIS points and uphill-specific roller-skiing performance than DP-TT; (3) laboratory performance indices in running and roller-ski skating showed large to very large correlations with distance FIS points and roller-skiing performance; and (4) laboratory performance indices obtained in running tended to be more strongly correlated with distance FIS points and overall and uphill-specific roller-skiing performance than corresponding values obtained in roller-ski skating.

Both field-based tests (RUN-TT and DP-TT) revealed moderate to large correlations with distance FIS points, as well as overall and terrain-specific roller-skiing performance. However, in contrast to the hypothesis, a stronger correlation was found between distance FIS points and RUN-TT performance than DP-TT performance, which is likely explained by the ability of uphill running tests to target the high demand for aerobic energy delivery in XC skiing, as well as greater involvement of muscle mass within this exercise modality [1, 28]. This is further supported by the associations between laboratory performance indices and the two field-based tests, in which peak speed and VO_2max_ in running were more strongly correlated with RUN-TT performance than DP-TT performance. Moreover, the present study found a strong correlation between RUN-TT and performance in uphill terrain of the roller-ski skating competition and RUN-TT was also included in both equations best predicting XC skiing and roller-skiing performance.

The abovementioned findings are somewhat in contrast to previous studies in XC skiing that have demonstrated higher relevance for short DP tests in the laboratory over tests performed in running or diagonal while classical roller-skiing [7, 8]. The reason for these conflicting findings is not known but could be related to differences in the methodology and heterogeneity of groups and the use of field versus laboratory tests. However, because of the longer duration of the RUN-TT, it is reasonable to suggest that a relatively short uphill DP-TT, as used here, would be more related to sprint XC skiing performance, targeting anaerobic endurance, as well as upper-body strength and high-speed capacities to a greater extent [1]. This is further supported by the strong correlations found between DP-TT performance and body mass as well as absolute VO_2max_ values, previously shown more related to sprint XC skiing performance [29].

In general, the present findings imply that simple field-based tests can be used as valid predictions of XC skiing and roller-skiing performance in a heterogeneous group of male XC skiers. The strength of implementing such tests is their high ecological validity combined with less need for expensive laboratory equipment and trained test personnel. Notably, the use of running field tests is further strengthened by the reduced influence of external factors (e.g., wind, temperature, and friction) compared to roller-skiing tests and particularly on-snow skiing tests. However, all field-based tests are to some extent influenced by external factors compared to laboratory tests, leading to lower test-retest reliability, in addition to providing less insights into the underlying mechanisms of different training adaptations.

Although there were no differences between RUN-TT and DP-TT in predicting performance in intermediate terrain of the roller-ski skating competition, there was a stronger correlation between DP-TT and performance in downhill terrain. Considering the significant correlation between body mass and DP-TT performance, this may be related to a higher level of upper-body strength and lean mass in these athletes. However, there was no correlations between body mass and performance in any terrain of the roller-ski skating competition. Another explanation might therefore be that upper-body strength is related to the skier’s ability to accelerate over hilltops due to a more pronounced upper-body propulsion, subsequently leading to higher speeds in downhill terrain. This is supported by findings in sprint XC skiing, in which high variations in speed at the end of uphill sections and transition into downhill sections were found [30]. Future studies should therefore investigate the role of both laboratory- and field-based tests in predicting micro-pacing patterns between skiers of different performance levels.

All performance indices in the laboratory, such as TTE, peak speed and VO_2max_ in a sport-specific (roller-ski skating) and non-specific (running) mode, showed large to very large correlations with distance FIS points as well as overall and terrain-specific performance in the roller-ski skating competition. However, performance indices in the laboratory were more strongly correlated with overall and terrain-specific performance than the two field-based tests, which are likely explained by more standardized test conditions and reduced influence of external factors. The strong correlations between VO_2max_ and overall and terrain-specific roller-skiing performance in this heterogeneous group of XC skiers were as expected and support the high demand for aerobic energy delivery in XC skiing [1, 28]. Moreover, the stronger correlations found between performance indices and performance in uphill versus intermediate and downhill terrain agree with previous findings [10, 12, 15], emphasizing the relevance of these laboratory-based tests for targeting the development of uphill-specific performance in XC skiers.

Furthermore, correlations between laboratory performance indices and XC skiing performance tended to be stronger for running than for roller-ski skating. These findings are coincided with the abovementioned field tests and the two equations best predicting XC skiing and roller-skiing performance, and the fact that most of the skiers reached their VO_2max_ in running. The reason for this is unclear, but it may be that the relatively young athletes in the present study have not yet developed their technical skills and specific endurance needed to achieve a high VO_2peak_/VO_2max_ ratio. However, the skiers reached a VO_2peak_/VO_2max_ ratio of ~95%, which is comparable to the ratios reported among elite XC skiers [1]. Moreover, XC skiers typically perform ~30–40% of their training in running during the preparation period and thus, develop high endurance capacities in running [1, 4, 5]. Overall, the present findings emphasize the importance of using performance indices such as TTE and peak speed in the laboratory while monitoring the training process of XC skiers, and test values obtained in running may be particularly relevant in heterogeneous groups of XC skiers.

All correlations of physiological responses, such as %VO_2max_, %HR_max_, and blood lactate levels during submaximal speeds in running and roller-ski skating, showed large to very large correlations with overall and terrain-specific performance. This indicates that the submaximal speeds were less demanding for the best-performing skiers and highlights the relevance of using simply measured variables, such as submaximal HR while monitoring XC skiers. However, gross efficiency in roller-ski skating showed no significant relationship with XC skiing performance, which is in contrast to previous findings [10, 16]. These conflicting findings might be explained by the fact that aerobic energy delivery is the main determining factor distinguishing performance in this group of XC skiers. However, gross efficiency might demonstrate different relationships with performance among more homogenous groups of elite XC skiers.

### Methodological considerations

While this study examined associations between laboratory- and field-based tests and XC skiing and roller-skiing performance, this only provides information about the validity of tests within this group of skiers at a given time-point. Correlation analysis does not establish cause-effect-relationships, and future studies should therefore investigate within-athlete development and how these tests are able to detect training-induced changes over longer timescales. Future studies should also aim to better understand the test-retest reliability of laboratory and field-based tests in XC skiing. Another potential limitation of the present design was the use of a roller-ski skating competition to determine overall and terrain-specific performance instead of an actual on-snow competition. However, the roller-ski skating competition was strongly correlated with distance FIS points, and the findings on performance predictions for different terrains are likely relevant for both on-snow XC skiing and roller-skiing performance. Although the athletes used the same type of roller-skis during the roller-ski skating competition and DP-TT, variations in roller resistance between skis may have influenced the current findings. It should also be noted that the distance FIS points represent performances from the previous competitive season, whereas all laboratory- and field-based tests were performed in the preparation period leading up to the upcoming season. Lastly, future studies including female skiers should examine if the different laboratory- and field-based tests demonstrate similar associations as found for the male skiers in the current study. It could be speculated that sex-specific differences in the validity of both laboratory- and field-based tests could be present due to differences in competition speeds, sub-technique selection and physiological, as well as anthropological characteristics between male and female XC skiers [1]. For example, Stöggl et al. [31] has previously shown that uphill performance have the strongest relationships to overall performance among female skiers, whereas performance in flat terrain seems more important for overall performance among male skiers.

## Conclusions and practical applications

The present findings suggest that both laboratory- and field-based tests provide valid predictions of XC skiing and roller-skiing performance in relatively heterogeneous groups of male XC skiers. Interestingly, both laboratory- and field-based tests in running tended to be more strongly correlated with XC skiing performance than corresponding values obtained in technique-specific modalities on roller skis. This might reflect the fact that aerobic energy delivery is the main determining factor distinguishing performance among athletes at this age and performance level. However, more sophisticated and mode-specific testing might be required for more homogenous groups of elite XC skiers. Altogether, the current data provide a novel understanding of how different laboratory- and field-based tests used to monitor training adaptations, predict performance in heterogenous groups of XC skiers. This is essential for coaches and athletes’ aiming to optimize the design and systematic use of tests to finetune their training process.

## Supporting information

S1 File Raw data. (XLSX)
